# Contextual and combinatorial structure in sperm whale vocalisations

**DOI:** 10.1038/s41467-024-47221-8

**Published:** 2024-05-07

**Authors:** Pratyusha Sharma, Shane Gero, Roger Payne, David F. Gruber, Daniela Rus, Antonio Torralba, Jacob Andreas

**Affiliations:** 1 Computer Science and Artificial Intelligence Laboratory, Massachusetts Institute of Technology, Cambridge, MA USA; 2 Project CETI, New York, NY USA; 3 Department of Biology, Carleton University, 1125 Colonel By Drive, Ottawa, ON K1S 5B6 Canada; 4 The Dominica Sperm Whale Project, Roseau, Dominica; 5 Baruch College and The Graduate Center, City University of New York, New York, NY USA

**Keywords:** Marine mammals, Animal behaviour, Marine biology

## Abstract

Sperm whales (*Physeter macrocephalus*) are highly social mammals that communicate using sequences of clicks called codas. While a subset of codas have been shown to encode information about caller identity, almost everything else about the sperm whale communication system, including its structure and information-carrying capacity, remains unknown. We show that codas exhibit contextual and combinatorial structure. First, we report previously undescribed features of codas that are sensitive to the conversational context in which they occur, and systematically controlled and imitated across whales. We call these rubato and ornamentation. Second, we show that codas form a combinatorial coding system in which rubato and ornamentation combine with two context-independent features we call rhythm and tempo to produce a large inventory of distinguishable codas. Sperm whale vocalisations are more expressive and structured than previously believed, and built from a repertoire comprising nearly an order of magnitude more distinguishable codas. These results show context-sensitive and combinatorial vocalisation can appear in organisms with divergent evolutionary lineage and vocal apparatus.

## Abstract (web summary)

Sperm whales use sequences of clicks to communicate. Here, the authors show that these vocalizations are significantly more complex than previously believed-the “sperm whale phonetic alphabet" has both combinatorial structure and call modulation dependent on the conversational context.

## Introduction

The social complexity hypothesis^1,2^ posits that animals in complex societies—featuring coordination, distributed decision-making, social recognition, and social learning of cultural strategies^3–6^—require complex communication systems to mediate these behaviours and interactions^1,7^. In humans, communication plays a particularly large role in complex social behaviours like strategising and teaching^8–10^. These behaviours require the ability to generate and understand a vast space of possible messages. For example, the sentence ‘Let’s meet next to the statue of Claude Shannon on the fifth floor at 3 pm’ picks out a specific action at a specific place and time from an enormous space of possible activities. This ability to generate complex messages, in turn, is supported by the fact that all known human languages exhibit contextual and combinatorial structure. (Throughout this paper, we use ‘context’ to denote conversational context—e.g., neighbouring codas—rather than behavioural context—e.g., diving—as is standard in the study of natural and formal languages.^11^) To enable efficient communication, the meaning of most human utterances is underspecified and derived in part from the conversation that precedes them^12^. To enable many distinct meanings to be communicated, humans access a large inventory of basic sounds (phonemes) by combining phonetic features like place of articulation, manner of articulation, then sequence phonemes to produce an unbounded set of distinct utterances^13–17^. Contextuality and combinatoriality, especially below the sequence level, have few analogues in communication systems outside of human language^18–22^. Understanding when and how aspects of human-like communication arise in nature offers one path toward understanding the basis of intelligence in other life forms.

**Fig. 1:**
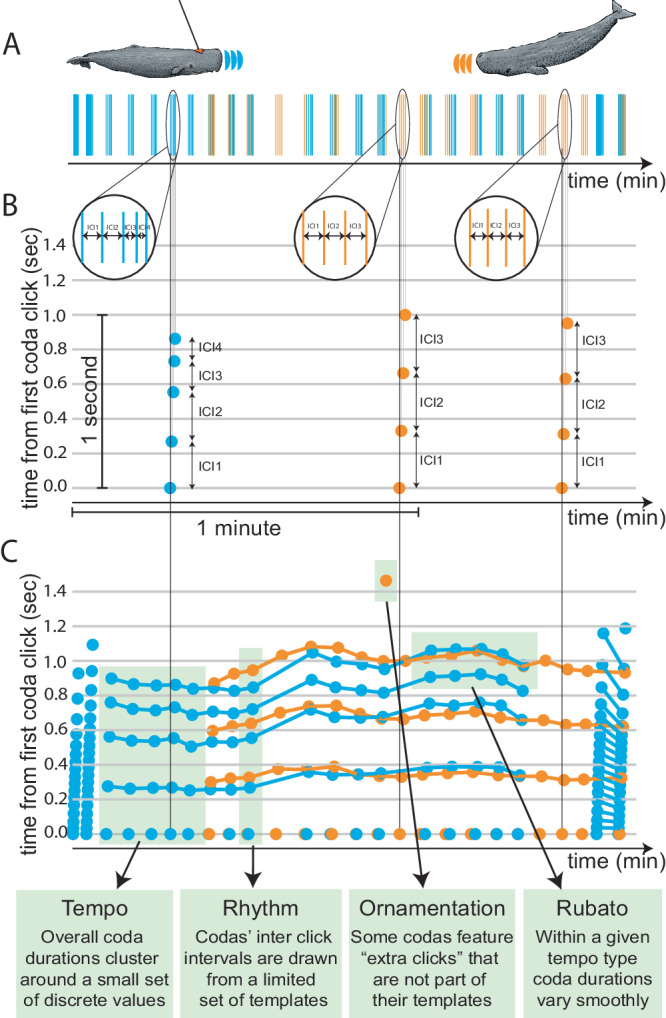
Exchange plot. Sperm whales communicate by producing sequences of clicks. **A** Shows a two-minute exchange between two whales (with clicks visualized in blue and orange respectively) from the Dominica Sperm Whale Dataset. **B** Projects these clicks over a time–time plot, in which the horizontal axis plots the time in the exchange at which a click occurs, and the vertical axis represents the time of the click from the first click in the coda. The vertical axis serves as a microscope over the horizontal axis, revealing the internal structure of each coda. **C** Shows a time–time visualisation for the entire two-minute exchange (with lines connecting matching clicks between adjacent codas), revealing complex, context-dependent variation in coda structure.

Cetaceans are an important group for the study of evolution and development of sophisticated communication systems^23^. Among cetaceans, long-term observational studies of sperm whales *(Physeter macrocephalus)* have described both a culturally defined, multi-level, matrilineal society^24^ and a socially transmitted communication system^25–27^. Sperm whales are known for complex social and foraging behaviour, as well as group decision-making^28^. They communicate using codes^29^: stereotyped sequences of 3–40 broadband clicks (see glossary in Table 1 for definitions). Codas are exchanged between whales when socialising or between long, deep, foraging dives^24^. To date, scientists have described the sperm whale communication system in terms of a finite repertoire of coda types, each defined by a characteristic sequence of inter-click intervals (ICIs) as seen in Fig. 1A. Coda-type repertoires can be defined manually or using automated clustering procedures, and have been used to delineate cultural boundaries among socially segregated but sympatric ‘clans’ of whales^25^ whose members differ in their behaviour^26,30,31^. Past research has identified around 150 discrete coda types globally, with 21 in the Caribbean. But there is an apparent contradiction between the social and behavioural complexity evinced by sperm whales and the comparative simplicity of a communication system with a fixed set of messages. This contradiction naturally raises the question of whether any additional, previously undescribed structure is present in sperm whale vocalisations.

**Table 1:** Glossary: definitions of previously used and newly introduced terminology

Notation	Description
Coda	A short burst of clicks with varying inter-click intervals generally less than two seconds in duration.
Inter-click interval (ICI)	The time difference between two consecutive clicks within a coda.
Absolute ICI	The absolute time difference between consecutive clicks in a given a coda as produced by the whale and recorded (see ref. ^25^).
Coda duration	The sum of a coda’s absolute ICIs.
Standardised absolute ICI	ICI normalised by the total duration of the containing coda. This conserves rhythm but discards tempo (see ref. ^25^).
Cumulative ICI	The absolute time difference between any given click and the first click of a coda as produced by the whale and recorded.
Standardised cumulative ICI	Relative ICI normalised by the total duration of the containing coda. This conserves the rhythm of the coda but discards the tempo. In codas normalised in this way, the last relative ICI is equal to 1 (compare to standardized absolute ICIs, which sum to 1).
Coda type	Categorical coda representation (primarily used in past work) obtained by clustering codas according to absolute ICI, which accounts for both rhythm and tempo simultaneously.
Rhythm type	The discrete category a coda is assigned to based on its characteristic sequence of standardized ICIs.
Tempo type	The discrete category a coda is assigned to based on its characteristic duration.
Exchange/chorus	Period of time where codas are made by more than a single whale (as in ref. ^54^).
Single-whale call sequence	A sequence of calls made by a given whale where every consecutive pair of calls occur within 8 seconds (twice the average response time) of each other.
Turn-taking	An exchange of codas involving alternating coda production. Also referred to as ‘adjacent’ codas, these are defined as next-in-sequence codas whose onset occurred within two seconds, but after the termination, of the initial coda (as in ref. ^33^).
Overlapping codas	An exchange of codas such that the next-in-sequence coda’s onset occurs after the onset, but before the termination, of the previous coda (as in ref. ^33^).
Ornament	‘Extra click’ appended to the end of a coda in a group of shorter codas. (For further details on the identification criterion, see Ornamentation section in the manuscript.)
Rubato	Gradual variation in duration across adjacent codas made by the same whale within the same rhythm and tempo type.

We first demonstrate that some coda structure is contextual: When analysing codas exchanged between whales, we observe fine-grained modulation of inter-click intervals relative to preceding codas, as well as modification of standard coda types via the addition of an extra click. We term these contextual features rubato and ornamentation. Next, we show that the coda repertoire has combinatorial structure: in addition to rubato and ornamentation, codas’ rhythms and tempos can independently be discretised into a small number of categories or types. We show that all four features are sensed and acted upon by participants in the vocal exchanges, and thus constitute deliberate components of the whale communication system rather than unconscious variation. Rhythm, tempo, rubato and ornamentation can be freely combined, together enabling whales to systematically synthesize an enormous repertoire of distinguishable codas. In a dataset of 8719 codas from the sperm whales of the Eastern Caribbean clan, this ‘sperm whale phonetic alphabet’ makes it possible to systematically explain observed variability in coda structure. While the communicative function of many codas remains an open question, our results show that the sperm whale communication system is, in principle, capable of representing a large space of possible meanings, using similar mechanisms to those employed by human sound production and representation systems (e.g., speech, text, Morse code, and musical notation).

## Results and discussion

### The dataset

For this study, we used the annotated coda dataset from The Dominica Sperm Whale Project (DSWP), the current largest sperm whale data repository. The recordings of the Eastern Caribbean 1 (EC-1) clan were used in the analysis, comprising 8719 codas making up 21 previously defined coda types. This dataset contains manually annotated coda clicks and extracted inter-click intervals in data recorded from various platforms and various recording systems between 2005 and 2018, including animal-worn, acoustic, biologging tags (DTags) deployed between 2014 and 2018. The EC-1 clan comprises 400 individuals. 42 tags were deployed on 25 different individuals in 11 different social units. Three of these are less-studied units, for which precise size estimates are not available. We conservatively estimate that at least 60 distinct whales are recorded in the DSWP dataset.

### Exchange plots: visualizing multi-whale calls

Codas, considered to be the basic units of sperm whale communication, consist of click groups generally less than two seconds in duration. Previous work has defined coda types and characterised coda repertoires by examining single codas outside the context in which they were produced. However, codas are not produced in isolation, but in interactive exchanges between two or more chorusing whales. Individual whales within a chorus tend to produce sequences of codas with a periodicity of approximately 4 seconds (see Supplementary Discussion Section 1). Across a chorus, interacting whales produce codas both alternately (i.e., turn-taking) or near-simultaneously (i.e., overlapping). Therefore, sperm whale vocalisations demonstrate complexity on two different timescales: a fine-grained time scale that determines the makeup of each individual coda, and a longer time scale that determines the overall structure of the interactive exchange across codas within a chorus.

We depict these vocalisations using a new visualisation we call an exchange plot (Fig. 1B, C). Both axes of this plot measure time: the horizontal axis shows the time elapsed since the beginning of a conversation, and the vertical axis shows the time since the onset of each coda. Exchange plots reveal the structure of codas in their interactive context, making it possible to observe both fine-grained differences between adjacent codas made by interacting whales, and long-range trends over the course of an exchange.

### Contextuality

Visualising whale vocalisations with an exchange plot, as in Fig. 1B, C, makes it apparent that characterising sperm whale interactions as sequences of fixed coda types overlooks a great deal of information. First, coda duration varies smoothly over the course of an exchange; variation in the duration of a whale’s codas is systematically imitated by interlocutors, even when coda-internal click spacing differs (Fig. 1C). Second, some ‘extra’ clicks in Fig. 1C appear at the end of codas that otherwise match their neighbours. We hypothesised that these smooth variations and extra clicks constitute perceptible and controllable features of codas independent of their discrete type, pointing toward a more complex sperm whale communication system with a greater information-carrying capacity than previously reported.

### Rubato: codas exhibit fine-grained duration variation in addition to discrete tempo

**Fig. 2:**
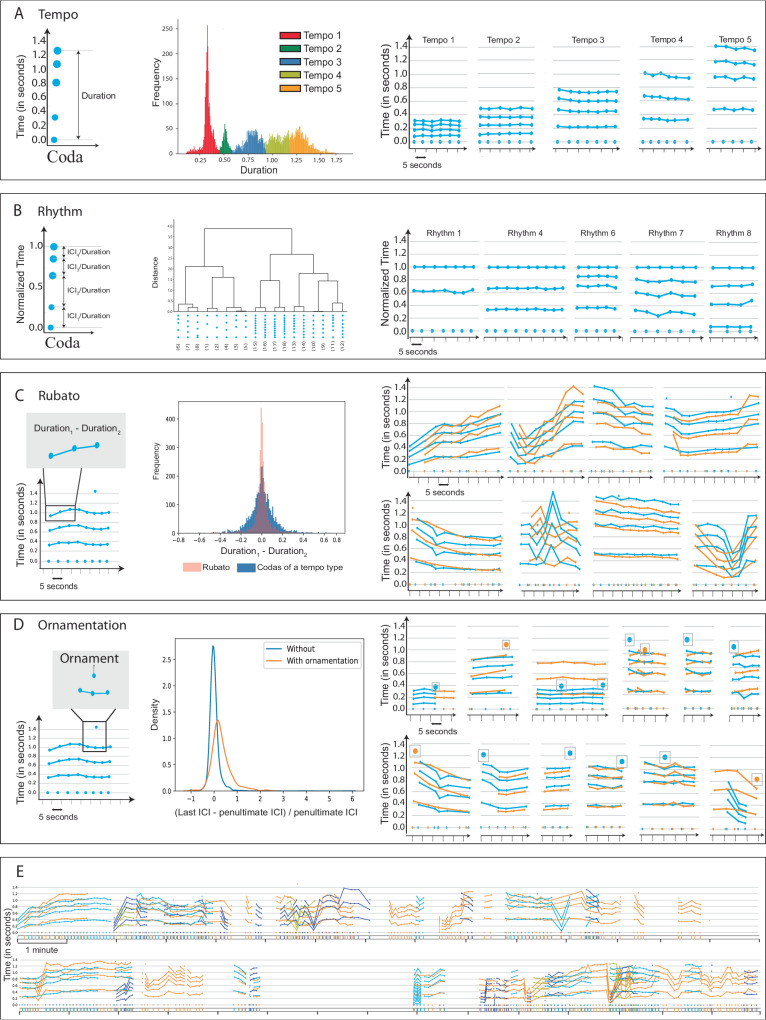
Combinatorial basis of coda production. Sperm whale codas were previously hypothesized to comprise 21 independent coda types. We show that this coda repertoire is built from two context-independent features (rhythm and tempo) and two context-sensitive features (rubato and ornamentation). **A** Tempo: (Left) The overall duration of a coda is the sum of its inter-click intervals. (Centre) Coda durations are distributed around a finite set of modes, which we call (tempo types). (Right) Snippets from exchange plots showing codas of different tempo types. **B** Rhythm: (Left) Normalising the vector of ICIs by the total duration returns a duration-independent coda representation, which we call rhythm. (Centre) Codas cluster around 18 rhythm types. (Right) Examples of normalised codas showing different rhythm types. **C** Rubato: (Left) Sperm whales slowly modulate coda duration across consecutive codas, a phenomenon we call rubato. (Centre) Rubato is gradual: adjacent codas have durations more similar to each other than codas of the same type from elsewhere in an exchange. (Right) Whale choruses with imitation of rubato represented in exchange plots. **D** Ornamentation: (Left) Some codas feature `extra clicks' (ornaments) not present in neighbouring codas that otherwise share the same ICIs. (Centre) A density plot showing the distribution of the ratio between final ICIs in ornamented codas versus unornamented codas. Ornamented codas have a significantly different ICI distribution compared to regular codas. (Right) Examples of ornaments in the DSWP dataset. **E** Thirty minutes of multi-whale choruses: Exchanges feature imitation of coda duration across whales, gradually accumulated changes in call structure, and rich contextual variability.

A coda’s duration is the sum of its inter-click intervals. While durations tend to cluster around a finite set of values (Fig. 2A), there remains substantial continuous variation within these clusters. Past work has described these differences as unexplained ‘within-type variation’ of categorical coda types^25,32^. However, the structured nature of this variation—which, as shown in Fig. 1C, is temporally correlated and imitated across whales—has never been previously documented. We demonstrate that variation in coda duration is not random: individual whales modulate coda durations smoothly over the course of multi-coda exchanges without necessarily imitating click counts or inter-click interval spacing. An example is depicted in Fig. 1C: one whale produces a sequence of codas smoothly varying in duration, while its interlocutor closely matches these changes in overall coda duration but not the number of clicks (this refines the finding in ref. ^33^ that overlapping codas were more likely to be of the same coda type than expected by chance: sometimes codas share a duration but not a discrete type). By analogy to the corresponding musical phenomenon, we call this behaviour rubato.

To show that rubato is not random variation, we first evaluated whether changes in duration are smooth by measuring whether codas are more similar to their neighbours than other codas of the same type. To do so, we computed the tempo drift between two codas from the same speaker, defined as the difference in coda durations (Fig. 2C). We computed the average absolute drift between (1) adjacent coda pairs and (2) random coda pairs of the same discrete coda type (according to its rhythm and tempo; see Section 5 for a discussion of rhythm and tempo and Supplementary Discussion Section 4 for additional details). We found that drift was significantly smaller between adjacent codas (0.05s on average) than would be expected under a null hypothesis that drift depends only on a coda’s discrete type (which would give a drift of 0.08s on average; test: permutation test (one-sided), *p* = 0.0001, *n* = 2593; see Supplementary Discussion Section 4.1 for details). Thus, within-type variation is context-dependent.

Second, we evaluated whether sequences of codas reflect longer-term trends. To do so, we collected coda triples of the same discrete coda type and measured the correlation between tempo drift across adjacent pairs. We found a significant positive correlation, compared to a null hypothesis that drift between adjacent pairs is uncorrelated (test: Spearman’s rank-order correlation (two-sided), *r*(2586) = 0.57, *p* < 0.0001, 95% CI = [0.54, 0.60], *n* = 2588). Thus, rubato is sustained across sequences of multiple codas.

Finally, we evaluated whether rubato is perceived and controlled by measuring whales’ ability to match their interlocutors’ coda durations when chorusing. We measured the average absolute difference in duration between (1) pairs of overlapping codas from different whales, and (2) pairs of non-overlapping codas of the same discrete coda type. Durations are significantly more closely matched for overlapping codas (0.099s on average) than would be expected under a null hypothesis that chorusing whales match only discrete coda type (which would give a drift of 0.129s on average) (test: permutation test (one-sided), *p* = 0.0001, *n* = 908; see Supplementary Discussion Section 4).

### Ornamentation: some clicks do not belong to standard tempo types

Figure 2D depicts an exchange consisting of one six-click coda, followed by a long sequence of five-click codas. The first five clicks of the initial 6-click coda closely match those of neighbouring codas: if the sixth click were removed, we would identify the first coda as having nearly the same inter-click intervals as its neighbours (and assign it to the same discrete coda type). While not previously described, ‘extra clicks’ of this kind occur in (4%) of the codas in the exchanges of Eastern Caribbean whales. Additional examples appear in Fig. 2D and Supplementary Discussion Section 5. We hypothesised that ‘extra’ clicks play a different role from the other clicks in the codas in which they appear: they do not determine discrete coda type. Instead, like rubato, they constitute an independent feature of the sperm whale vocalisation system. We call these extra clicks ornaments.

We define an ornament as the final click in a coda containing one more click than the nearest preceding or following coda within a window of ten seconds, with this interval being based on the average response time (Supplementary Discussion Section 1). To show that these ornaments play a distinct role from other clicks, we first computed the mean squared distance between each standardised, ornamented coda and the cluster centre of its assigned rhythm type. We then removed ornaments and computed mean squared distance between the standardised coda and the centres of rhythm clusters for adjacent codas produced by the same whale. The second quantity (0.0034 s^2^, reflecting a hypothesis that ornamented codas match their neighbours) is significantly smaller than the first (0.0053 s^2^, reflecting a null hypothesis that ornamented codas resemble other codas of the same type) (test: permutation test (one-sided), *p* = 0.002, *n* = 178). In other words, ornamented codas are less like other codas with a matching number of clicks, and more like neighbouring non-ornamented codas, if ornaments are modelled as a separate factor. To further verify that ornaments are distinct from other clicks, we compared ICIs (inter-click intervals) in ornamented vs. non-ornamented codas with the same number of clicks. We specifically compared the difference between the final two ICIs, normalised by the penultimate ICI, to reduce variance arising from rubato. This measurement exhibits a significant difference in distribution in ornamented vs non-ornamented codas test: Kolmogorov–Smirnov test (two-sample), *D*(150, 3688) = 0.51, *p* < 0.0001, 95% CI = [0.42, 0.62], *n* = (150, 3688), Fig. 2D.

Finally, ornaments are not distributed randomly but appear in distinctive positions in longer exchanges. Within a single whale’s call sequences (defined as a sequence of codas separated by no more than eight seconds), a significantly greater proportion of ornamented codas appear at the beginning of call sequences than unornamented codas (test: Fisher’s exact test (two-sided), odds ratio: 2.00, *p* = 0.0006). A significantly greater fraction of ornamented codas also appear at the end of call sequences compared to unornamented codas (test: Fisher’s exact test (two-sided), odds ratio: 1.71, *p* = 0.008). Moreover, ornaments are predictive of changes in multi-whale interactions. We define a ‘change in chorusing behaviour’ as one of three events: a following whale begins chorusing with a leading whale, pauses chorusing, or ceases vocalizing for the remainder of the exchange. Compared to unornamented codas, ornamented codas from the leading whale are disproportionately succeeded by a change in chorusing behaviour from the following whale (test: Fisher’s exact test (two-sided), odds ratio: 1.56, *p* = 0.009). Thus ornamentation, like rubato, appears to be perceived in multi-whale interactions.

### Combinatoriality

The existence of ornamentation and rubato features demonstrates that codas can carry more information, and have more complex internal structure than their discrete type alone would indicate. Instead, they result from a combinatorial coding system in which discrete type, ornamentation and rubato combine to realise individual codas. Based on these findings, we hypothesized that categorical coda types might themselves arise from a combinatorial process, with a simpler set of features explaining the prototypical ICI vector for each coda type.

During rubato, consecutive calls from a single-whale gradually vary coda duration while preserving the relative relationship of ICIs (Supplementary Discussion Section 2), suggesting that whales are capable of maintaining this relationship independent of its duration. Moreover, existing studies have noted that codas may also be discretely clustered according to standardised ICI^25,32,34^, a process that assigns codas with very different durations to the same cluster. Finally, some instances of chorusing involve whales producing codas with different ICIs (or different numbers of clicks) but matched durations. Together, these observations suggest that past inventories of discrete coda types (e.g., ref. ^35^) might specifically be interpretable in terms of two features: a normalised ICI category (which we term a coda’s rhythm) and a discrete duration category (independent of rubato, and which we term tempo). To validate this hypothesis, we measured (1) whether coda rhythms and tempos cluster around a discrete set of values, and (2) whether rhythm and tempo features are independently combinable (both with each other and with ornamentation and rubato features).

As shown in Fig. 2A, codas with the same duration may have different internal click spacing (and even different numbers of clicks) but still span the same amount of time from the first click to the last click. Performing kernel density estimation (KDE) on scalar coda durations from the DSWP dataset reveals five distinct modes in the distribution of durations (Fig. 2A), indicating that the number of realised coda durations is much smaller than the total number of identified coda types (Supplementary Discussion Section 3).

Across codas, the relative relationships between ICIs are often repeated even independent of tempo. For example, in Fig. 2A, note the existence of two five-click codas, one long and one short, but both characterised by the uniform spacing of the constituent ICIs. Past work has shown that these rhythms are reused; our analysis uses the 18 rhythm clusters proposed by ref. ^35^ (detailed breakdowns are given in Fig. 2B and in Supplementary Discussion Section 2).

**Fig. 3:**
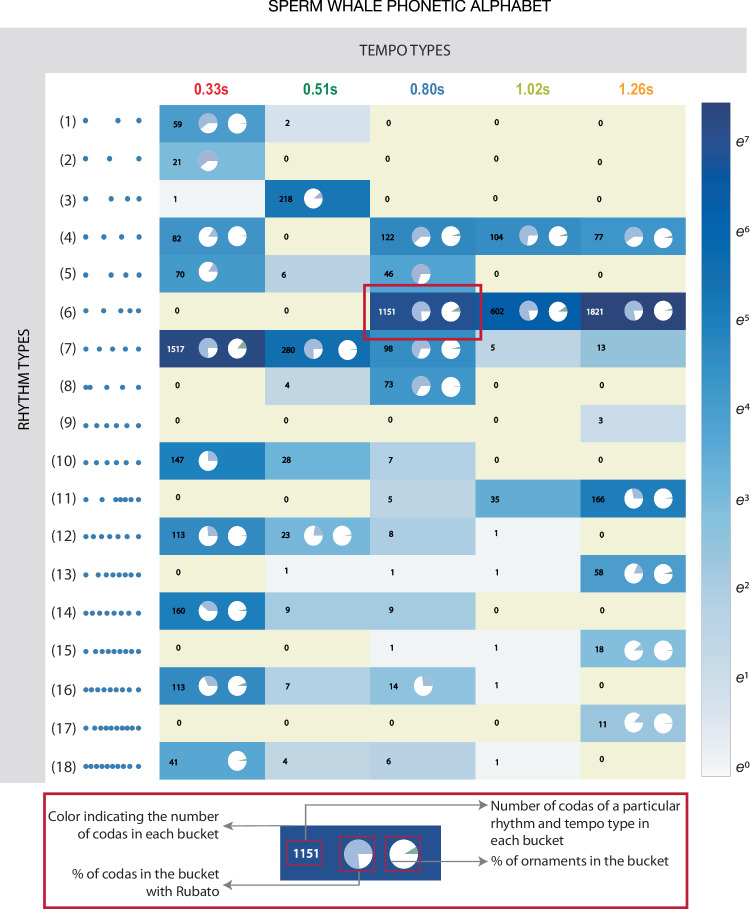
Sperm whale phonetic alphabet. Analogous to visualizations of the human phonetic repertoire, we propose a phonetic alphabet for sperm whales. Tempo types are plotted on the vertical axis, rhythm types are plotted on the horizontal axis, and the colour of each cell represents the number of occurrences of that rhythm/tempo combination in the DSWP dataset. Pie charts in each cell provide further information about the prevalence of rubato and ornamentation within each feature combination: the left pie shows the ratio of the number of codas that appear with rubato to those without, while the right pie shows the fraction of all ornaments that appear with that feature combination. While not all feature combinations are realised (as observed in human languages), sperm whale codas have a rich combinatorial structure with both discrete and continuous parameters and at least 143 combinations frequently realised (Supplementary Discussion Section 6).

Finally, to evaluate the combinability of these features, we computed the frequency with which each rhythm and tempo feature co-occurred in the DWSP dataset, as well as the frequency with which each combination appeared with ornamentation or rubato. Results are shown in Fig. 3. Each rhythm type appears with at least one tempo types and each tempo type appears with at least three rhythm types. Moreover, (22%) of these combinations can appear with or without rubato and ornamentation.

Like the International Phonetic Alphabet for human languages, this ‘Sperm Whale Phonetic Alphabet’ (Fig. 3) shows how a small set of axes of variation (place of articulation, manner of articulation, and voicedness in humans; rhythm, tempo, ornamentation, and rubato in sperm whales) give rise to the diverse set of observed phonemes (in humans) or codas (in sperm whales). As in human languages, not all theoretically realisable feature combinations are attested in the DSWP dataset, and some combinations are more frequent than others. As in human languages, most coda variation is discrete: though ICIs can vary continuously in principle, only specific patterns (associated with specific rhythms and tempos) are realised in practice. Supplementary Discussion Section 2 shows the full set of codas in the dataset, organised by rhythm, tempo, and the presence of rubato and ornamentation for each combination of rhythm and tempo. Notably, these factors of sub-coda variation exist alongside another combinatorial process—the sequential ordering of codas shown in Fig. 1E—in which codas of different types are combined in sequence to give rise to an even larger family of distinct vocalisations, reminiscent of the bi-level combinatorial structure of speech production in humans (see Supplementary Discussion Section 7).

Figure 3 also demonstrates that these vocalisations have a significantly greater information capacity than was previously known. Prior work identified 21 discrete coda types, and the system could be understood to have an information rate of at most 5 bits/coda. However, our analysis suggests that with 18 rhythms, 5 tempos, optional ornamentation, and three variations (increasing, decreasing or constant duration) in rubato, the information rate could be up to twice as large (details in Supplementary Discussion Section 6). The role of rubato within this coding system remains an important open question: it might be discrete (with some simpler inventory of contours explaining the patterns in Figs. 1C and 2C, as in the songs of birds^36–41^, and humpback whales *(Megaptera novaeangliae)*^42,43^). Or it might convey continuous-valued information, analogous to the orientation and duration features of the waggle dance in bees *(Apis sp.)*^44^.

### Limitations

Our study investigates the basic structural elements, and not the semantics, of the sperm whale communication system. As in several foundational papers on call structure in animal communication systems^42,45,46^, it provides no characterisation of call semantics and features no playback experiments. We believe our work provides a foundation for future research on the semantics of whale calls. However, this future research additionally requires interactive playback experiments with whales in the wild to ground hypotheses about the semantics and functional role of sperm whale vocalizations. In the absence of playback experiments establishing a causal relationship between the features and the meaning they communicate is challenging. It is necessary to have a deep understanding of the structure of the communication system enabling the creation of specific and unconfounded test stimuli prior to undertaking whale-in-the-loop experiments with potentially long-term effects on the population^47,48^, which in this case is extremely vulnerable^49^.

### Concluding remarks

Our results demonstrate that sperm whale vocalisations form a complex combinatorial communication system: the seemingly arbitrary inventory of coda types can be explained by combinations of rhythm, tempo, rubato, and ornamentation features. Sizable combinatorial vocalisation systems are exceedingly rare in nature; however, their use by sperm whales shows that they are not uniquely human, and can arise from dramatically different physiological, ecological, and social pressures.

These findings also offer steps towards understanding how sperm whales transmit meaning. In some organisms with combinatorial codes, such as honey bees *(Apis sp.)*, the constituent features of the code transparently encode semantics (e.g., direction and distance to food sources). Further research on sperm whale vocalisations may investigate if rhythm, tempo, ornamentation, and rubato function similarly, directly encoding whales’ communicative intents. Alternatively, one of the key differentiators between human communication and all known animal communication systems is duality of patterning: a base set of individually meaningless elements that are sequenced to generate a very large space of meanings. The existence of a combinatorial coding system-at either the level of sounds, sound sequences, or both-is a prerequisite for duality of patterning. Our findings open up the possibility that sperm whale communication might provide our first example of that phenomenon in another species.

## Methods

### Data collection and coda annotation

Data from The Dominica Sperm Whale Project were collected under scientific research permits from the Fisheries Division of the Government of Dominica. The field protocols for approaching, photographing, tagging, and recording sperm whales were approved by either the University Committee on Laboratory Animals of Dalhousie University, Canada; the Animal Welfare and Ethics Committee of the University of St Andrews, Scotland; or Aarhus University, Denmark; and sometimes several or all of these across years.

Social units of female and immature sperm whales were located and followed in an area that covered approximately 2000 squared kilometres along the entire western coast of the Island of Dominica (N15.30 W61.40) between 2005 and 2018.

Codas were recorded using one of several recording setups: In 2005, we used a Fostex VF-160 multitrack recorder (44.1 kHz sampling rate) and a custom-built towed hydrophone (Benthos AQ-4 elements, frequency response: 0.1–30 kHz) with a filter box with high-pass filters up to 1 kHz resulting in a recording chain with a flat frequency response across a minimum of 2–20 kHz. No recordings were made during the short 2006 season. In the 2007, 2009, 2011, 2016, and 2017 seasons, we used a Zoom H4 portable field recorder (48 kHz sampling rate) and a Cetacean Research Technology C55 hydrophone (frequency response: 0.02–44 kHz) and no filters. During the 2008, 2010, 2012, 2015, and 2018 seasons, we used a custom-built towed hydrophone (Benthos AQ-4 elements, frequency response: 0.1–30 kHz) with a filter box with high-pass filters up to 1 kHz resulting in a recording chain with a flat frequency response across a minimum of 2–20 kHz. This was connected to a computer-based recording system as a part of the International Fund for Animal Welfare’s (IFAW) LOGGER software package (48 kHz sampling rate) or PAMGUARD (minimum 48 kHz sampling rate)^50^.

In addition, recordings were also made through the deployment of animal-borne sound and movement tags (DTag generation 3, Johnson and Tyack 2003). Tagging was undertaken between 2014 and 2018 on an 11-meter rigid-hulled inflatable boat (RHIB). Tags were deployed from a 9-meter, hand-held, carbon fiber pole, and were attached to the whales using four suction cups. DTags record two-channel audio at 120 kHz with a 16-bit resolution, providing a flat (±2 dB) frequency response between 0.4 and 45 kHz. Pressure and acceleration were sampled at a rate of 500 Hz with a 16-bit resolution, and were decimated to 25 Hz for analysis. DTag analysis was conducted using custom scripts in Matlab 2015b (The Mathworks, Inc., MA, USA). The variation in the frequency responses and sampling rates of the recording systems used did not affect our ability to record clean signals for both the coda and echolocation clicks produced by sperm whales, and as a result, the temporal patterning of clicks used in this analysis.

Whales, including the tagged whales, were identified through photographs of the trailing edge of their tails^51^. Identifications were used to ensure that only recordings from one of the two sympatric clans (EC-1, the Eastern Caribbean Clan) were included in the analysis to control for any differences in repertoires between vocal clans^27^.

To define the temporal structure of the codas recorded, absolute inter-click intervals were measured as in ref. ^49^, using either custom-written Matlab tools and Rainbow Click Software (all years before 2014) or CodaSorter a custom-written tool (K. Beedholm, Marine Bioacoustics Lab, Aarhus University) in LabView (National Instruments, TX, USA). CodaSorter allows users to playback audio at various speeds and manually mark detected clicks as belonging to a specific coda. Estimates for each click for the angle of arrival, channel delay, centroid frequency, and inter-pulse interval (IPI, the time between the onset of the first pulse and the onset of the next pulse in the multi-pulse structure of sperm whales clicks^52^) allowed for determining if the codas were produced by tagged whales or non-focal animals; and to ensure that, on days in which multiple tags were deployed, codas recorded by different tags were not double-counted. Photo-identification supported this process by identifying which whales were present and associated with the tagged whales at each surfacing.

There are two components to the dataset: Dataset 1, a large set of all 8719 codas that are annotated with information on their inter-click intervals; and Dataset 2, a smaller set of 3948 codas, which were recorded from animal-borne DTags, which remain in temporal order and are additionally annotated with information of the absolute time in the day of the first click of each of the codas and their associated speaker identities across the bouts. Experiments that do not require contextual information (those discussing the context-independent features of rhythm and tempo) use Dataset 1, whereas those requiring information about the relative ordering of the codas and their speaker IDs (those discussing the context-sensitive features of rubato and ornamentation), use Dataset 2. In both cases, rare, long codas were excluded from analysis (greater than 10 clicks, less than 5% of all codas recorded).

### Additional discussion of statistical tests

Comparisons of coda durations (either with adjacent codas, when studying rubato, or with overlapping codas, when studying all features in the context of chorusing behaviour) use permutation tests to avoid making distributional assumptions about durations of codas and their absolute differences, some of which have non-normal distributions. All permutation tests are computed over 10,000 random resamplings of the data without replacement. Evaluation of Rubato additionally uses Spearman rank-correlation tests to measure longer-range trends across coda triplets (again based on initial observations that these trends appeared to be non-linear). Comparisons of coda-internal structure (e.g., durations of penultimate ICIs in ornamented codas) use Kolmogorov–Smirnov tests, as we are interested only in distributional differences rather than orderings of mean durations. Finally, measurements of changes in vocalization behaviour following Rubato use Fisher’s exact test to compare proportions of these changes in different vocal contexts.

### Reporting summary

Further information on research design is available in the Nature Portfolio Reporting Summary linked to this article.

### Supplementary information

Supplementary Information

Peer Review File

Reporting Summary

## Data Availability

All data generated and in this study has been deposited on GitHub at https://github.com/pratyushasharma/sw-combinatoriality/tree/main/data^53^.

## Code availability

Custom scripts used for this study are available at https://github.com/pratyushasharma/sw-combinatoriality^53^.

## References

[1] Freeberg TM, Dunbar RIM, Ord TJ (2012). Social complexity as a proximate and ultimate factor in communicative complexity. Philos. Trans. R. Soc. Lond. B Biol. Sci..

[2] Peckre L, Kappeler PM, Fichtel C (2019). Clarifying and expanding the social complexity hypothesis for communicative complexity. Behav. Ecol. Sociobiol..

[3] Dunbar RIM (2009). The social brain hypothesis and its implications for social evolution. Ann. Hum. Biol..

[4] Dunbar RIM (1993). Coevolution of neocortical size, group size and language in humans. Behav. Brain Sci..

[5] Byrne RW, Whiten A (1990). Machiavellian intelligence: social expertise and the evolution of intellect in monkeys, apes, and humans. Behav. Philos..

[6] Taborsky B, Oliveira RF (2012). Social competence: an evolutionary approach. Trends Ecol. Evol..

[7] Freeberg TM (2006). Social complexity can drive vocal complexity: group size influences vocal information in Carolina chickadees. Psychol. Sci..

[8] Hauser, M. D. *The Evolution of Communication* (MIT Press, 1996).

[9] Jackendoff, R. *Foundations of Language: Brain, Meaning, Grammar, Evolution* (Oxford Univ. Press, UK, 2002). 10.1017/s0140525x03000153 15377127

[10] Hauser MD, Chomsky N, Fitch WT (2002). The faculty of language: What is it, who has it, and how did it evolve?. Science.

[11] Chomsky, N. *Syntactic Structures* (The Hague, Mouton, 1957).

[12] Grice, H. P. Logic and Conversation. In *Speech acts*. Syntax and semantics, Vol. 3 (eds Cole, P. & Morgan, J. P.) (Seminar Press, 1975).

[13] Panini. *Ashtadhyayi, Digital Library of India* (520).

[14] Hockett CD (1960). The origin of speech. Sci. Am..

[15] Chomsky, N. *Language and Mind* (Cambridge Univ. Press, UK, 1968).

[16] Lieberman, P. *The Biology and Evolution of Language* (Harvard University Press, 1984).

[17] Fitch WT (2000). The evolution of speech: a comparative review. Trends Cogn. Sci..

[18] Yip MJ (2006). The search for phonology in other species. Trends Cogn. Sci..

[19] Collier K, Bickel B, van Schaik CP, Manser MB, Townsend SW (2014). Language evolution: syntax before phonology?. Proc. R. Soc. B Biol. Sci..

[20] Bowling DL, Fitch WT (2015). Do animal communication systems have phonemes?. Trends Cogn. Sci..

[21] Engesser S, Townsend SW (2019). Combinatoriality in the vocal systems of nonhuman animals. Wiley Interdiscip. Rev. Cogn. Sci..

[22] Fitch WT (2019). Sequence and hierarchy in vocal rhythms and phonology. Ann. N. Y. Acad. Sci..

[23] King SL, Connor RC, Montgomery SH (2022). Social and vocal complexity in bottlenose dolphins. Trends Neurosci..

[24] Whitehead H (2004). Sperm whales: social evolution in the ocean. Choice.

[25] Rendell LE, Whitehead H (2003). Vocal clans in sperm whales (Physeter macrocephalus). Proc. Biol. Sci..

[26] Cantor M, Whitehead H (2015). How does social behavior differ among sperm whale clans?. Mar. Mamm. Sci..

[27] Gero S, Bøttcher A, Whitehead H, Madsen PT (2016). Socially segregated, sympatric sperm whale clans in the Atlantic Ocean. R. Soc. Open Sci..

[28] Whitehead H (2016). Consensus movements by groups of sperm whales. Mar. Mamm. Sci..

[29] Watkins WA (1977). Sperm whale codas. J. Acoust. Soc. Am..

[30] Marcoux M, Whitehead H, Rendell L (2007). Sperm whale feeding variation by location, year, social group and clan: evidence from stable isotopes. Mar. Ecol. Prog. Ser..

[31] Whitehead H, Rendell L (2004). Movements, habitat use and feeding success of cultural clans of South Pacific sperm whales. J. Anim. Ecol..

[32] Antunes R (2011). Individually distinctive acoustic features in sperm whale codas. Anim. Behav..

[33] Schulz TM, Whitehead H, Gero S, Rendell L (2008). Overlapping and matching of codas in vocal interactions between sperm whales: insights into communication function. Anim. Behav..

[34] Moore KE, Watkins WA, Tyack PL (1993). Pattern similarity in shared codas from sperm whales (Physeter catodon). Mar. Mamm. Sci..

[35] Gero S, Whitehead H, Rendell L (2016). Individual, unit and vocal clan level identity cues in sperm whale codas. R. Soc. Open Sci..

[36] Catchpole, C. K. & Slater, P. J. B. *Bird Song* 2nd edn (Cambridge Univ. Press, UK, 2008).

[37] Sasahara K, Cody ML, Cohen D, Taylor CE (2012). Structural design principles of complex bird songs: a network-based approach. PLoS ONE.

[38] Engesser S, Crane JMS, Savage JL, Russell AF, Townsend SW (2015). Experimental evidence for phonemic contrasts in a nonhuman vocal system. PLoS Biol..

[39] Suzuki TN, Matsumoto YK (2022). Experimental evidence for core-merge in the vocal communication system of a wild passerine. Nat. Commun..

[40] Suzuki TN, Wheatcroft D, Griesser M (2017). Wild birds use an ordering rule to decode novel call sequences. Curr. Biol..

[41] Suzuki TN, Wheatcroft D, Griesser M (2016). Experimental evidence for compositional syntax in bird calls. Nat. Commun..

[42] Payne RS, McVay S (1971). Songs of humpback whales. Science.

[43] Allen JA, Garland EC, Dunlop RA, Noad MJ (2019). Network analysis reveals underlying syntactic features in a vocally learnt mammalian display, humpback whale song. Proc. Biol. Sci..

[44] Frisch KV (1967). The dance language and orientation of bees. J. Anim. Ecol..

[45] Whiten A (1999). Cultures in chimpanzees. Nature.

[46] Simpson J, von Frisch K (1969). The dance language and orientation of bees. J. Anim. Ecol..

[47] Herbinger I, Papworth S, Boesch C, Zuberbühler K (2009). Vocal, gestural and locomotor responses of wild chimpanzees to familiar and unfamiliar intruders: a playback study. Anim. Behav..

[48] Harris JBC, Haskell DG (2013). Simulated birdwatchers’ playback affects the behavior of two tropical birds. PLoS ONE.

[49] Gero S, Whitehead H (2016). Critical decline of the eastern Caribbean sperm whale population. PLoS ONE.

[50] Gillespie D (2009). PAMGUARD: Semiautomated, open source software for real time acoustic detection and localization of cetaceans. J. Acoust. Soc. Am..

[51] Arnbom, T. *Individual Photographic Identification: a Key to the Social Organization of Sperm Whales, Thesis (M.Sc.)* (Memorial University of Newfoundland, 1987).

[52] Møhl B, Wahlberg M, Madsen PT, Heerfordt A, Lund A (2003). The monopulsed nature of sperm whale clicks. J. Acoust. Soc. Am..

[53] Sharma, P. pratyushasharma/sw-combinatoriality: sw-combinatoriality (sw-combinatoriality). *Zenodo*. 10.5281/zenodo.10817697 (2024).

[54] Ravignani A, Bowling DL, Fitch WT (2014). Chorusing, synchrony, and the evolutionary functions of rhythm. Front. Psychol..

