# Supplementary material for: Contextual and combinatorial structure in sperm whale vocalisations
Source: Nat Commun. 2024 May 7;15:3617. doi: 10.1038/s41467-024-47221-8 (PMC11076547; doi:10.1038/s41467-024-47221-8)
Supplement: Supplementary file 1 — Supplementary Information [file 41467_2024_47221_MOESM1_ESM.pdf]

Supplementary Discussion: Contextual and Combinatorial Structure in Sperm Whale Vocalisations

1 Preliminaries

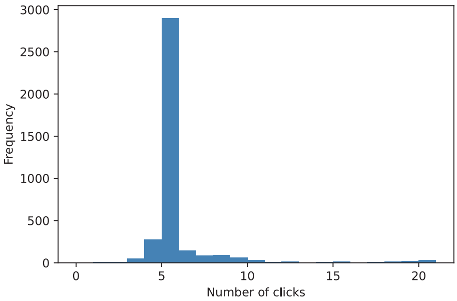

Supplementary Fig. 1 Frequency distribution of codas based on the total number of clicks in the coda for the Dominica Sperm Whale Project Dataset. Sperm whales of the Eastern Caribbean Clan-1 predominantly produce 5-click codas.

Consider a coda $\mathbf{c}$ consisting of five clicks occurring at times $[t_1, t_2, t_3, t_4, t_5]$. We may represent this coda as a vector of four *inter-click intervals* c, writing:

$$\mathbf{c} = [c_1, c_2, c_3, c_4] = [t_2 - t_1, t_3 - t_2, t_4 - t_3, t_5 - t_4] \ . \tag{1}$$

An example is depicted in Fig. 1(B) of the main paper. This coda consists of two long ICIs followed by two short ICIs. The coda may also be represented by its *cumulative inter-click intervals*:

$$\mathbf{c}_{REL} = [t_2 - t_1, t_3 - t_1, t_4 - t_1, t_5 - t_1] \ . \tag{2}$$

The exchange plot visualization plots the ith click of the coda such that,

$$(x, y) = (t_i, t_i - t_1) \ . \tag{3}$$

(75%) of the codas used by sperm whales of the Eastern Caribbean clan are 5-click codas. Fig. 1 shows the frequency distribution of click count.

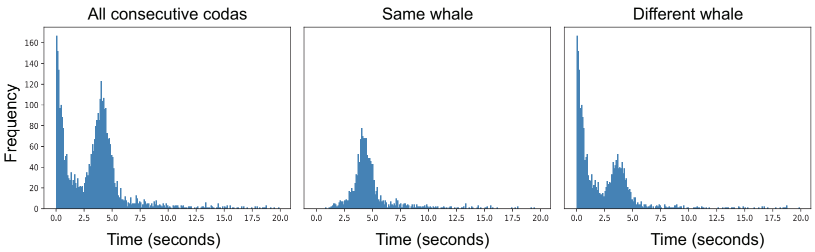

Supplementary Fig. 2 Response time: In the EC1 clan, consecutive codas are produced such that they either overlap with one another (response time ≈ 0) or after a time period of four seconds. *(Left)* The frequency distribution of the response time between consecutive codas. *(Center)* The frequency distribution of the response time if the consecutive codas were made by the same speaker, denoting the four-second isochronous periodicity of the coda production of an individual whale; and *(Right)* The frequency distribution of response time when consecutive codas are produced by different whales. The peak at $t = 0$ corresponds to overlapping codas, and the peak at $t \approx 4$ corresponds to codas produced during turn-taking.

The periodicity of codas produced by a single whale is fairly isochronous with speakers making codas with an interval of approximately four seconds. During exchanges between whales or in chorus, the inter-coda interval is bimodal, with some codas being overlapped by their interlocutor and others being produced in sequence at an interval of approximately four seconds (Fig. 2).

2 Rhythm

A coda's rhythm is determined by its characteristic sequence of standardized ICIs. We use the rhythm clusters shown in Fig. 3, reported by [1]. While [1] has provided intrinsic (within-coda) evidence that rhythm types are clustered, this clustering is also reflected at the exchange level, between adjacent codas. We present two pieces of temporal evidence that indicate the need for an independent treatment of the overall duration of the coda from its underlying rhythm.

2.1 Transition dynamics of consecutive calls

The plot in Fig. 4 shows the probability of transitioning between codas of various rhythm types by a single whale. Transitions between adjacent codas of the same rhythm type are significantly more likely than cross-type transitions, making up (82%) of all transitions. This is also clear from exchange plots, in Fig. 1 of the main paper, in which a given whale can be seen to maintain its rhythm in the course of producing consecutive calls. A high probability of transitioning into the same rhythm cluster in consecutive turns indicates that discovered clusters are also coherent in time.

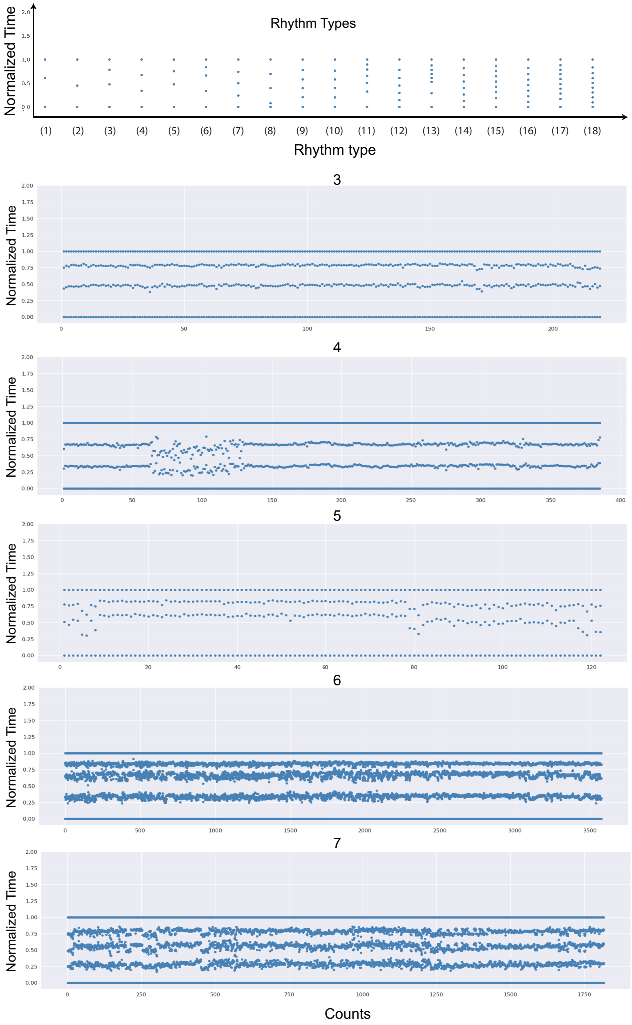

Supplementary Fig. 3 *(Top)* The set of coda rhythm types denoted by the mean standardized coda in each of the clusters. *(Bottom)* The corresponding set of codas of rhythm types 3 to 7.

2.2 Overlapping codas with distinct rhythm types still match overall durations

Sometimes, overlapping codas exhibit different rhythm types (as shown in Fig. 5) and even different numbers of clicks. For example, (14%) of overlapped codas involve one 4-click coda and one 5-click coda. Even when rhythm type is not matched, duration often is matched over the course of an exchange: the difference between overlapping coda durations is on average 0.104 seconds. Under a null hypothesis that this duration matching is fully explained by the overlapping codas' discrete types, we would expect a difference in durations of

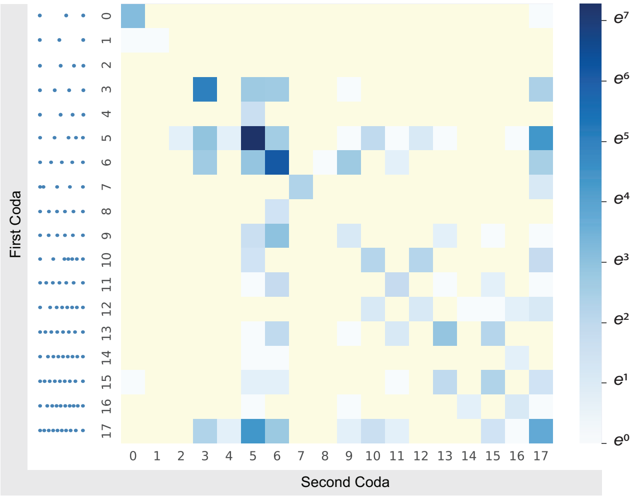

Supplementary Fig. 4 Frequencies of transitions between different rhythm types.

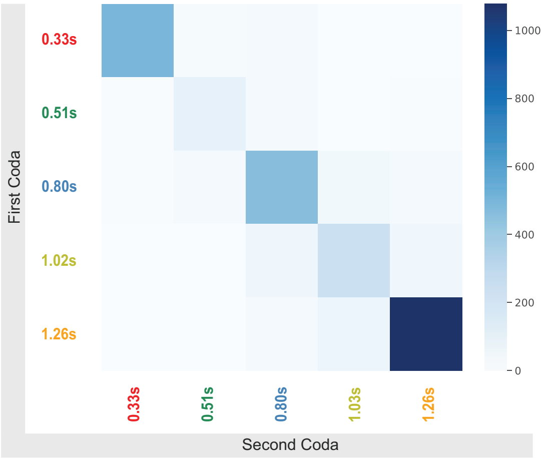

Supplementary Fig. 5 Frequencies of transitions between different tempo types. The matrix is diagonally dominant.

0.134 seconds, which is significantly larger (test: permutation test (one-sided), $p = 0.0001$, $n = 248$, performed by randomly resampling durations within each discrete coda type without replacement). Thus, rhythm and tempo features can be independently combined:[1] tempo can be imitated even while holding different rhythm types constant.

[1]We emphasize that this does not imply that rhythm and tempo are statistically independent: indeed, codas with specific rhythm types are more likely to have certain tempo types than others.

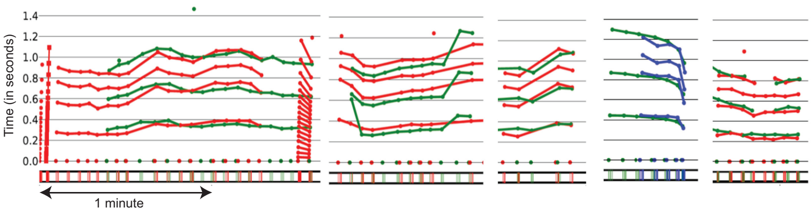

Supplementary Fig. 6 A set of exchange plots showing choruses between pairs of sperm whales. Here, whales exchange overlapping codas of different rhythm types while matching duration. This demonstrates independent control of rhythm and duration during coda production.

3 Tempo

A coda's tempo is a discretised version of its overall duration.

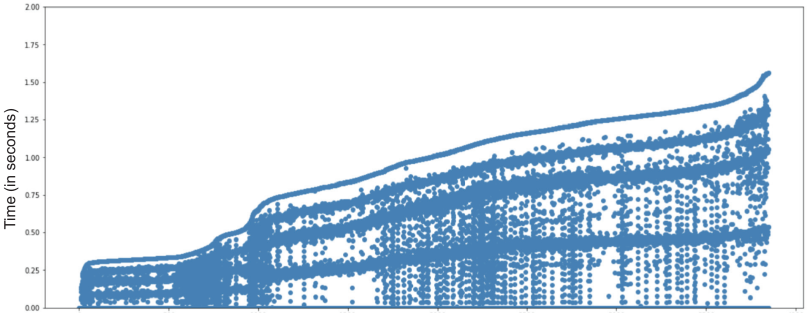

Supplementary Fig. 7 All DSWP codas visualized in increasing order of overall duration. The empirical distribution of the duration cluster around a set of small modes as seen in Fig. 2(B) of the main manuscript.

3.1 Clustering

We obtained the empirical distribution of coda durations from the DSWP dataset, then estimated the probability density function of the duration using a kernel density estimator (KDE) with a Gaussian kernel function. The bandwidth of the estimator (h=0.035) was chosen such that it minimizes the mean integrated squared error. The empirical distributions of codas cluster around a small set of modes as seen in Fig. 2(B) of the main manuscript. Any further reduction in the bandwidth causes the estimator to produce a larger set of modes in regions with insufficient data points, such as regions with duration (< 0.1) seconds and duration (> 1.5) seconds. This estimated distribution discovers a set of 5 peaks with values at $[0.33, 0.51, 0.8, 1.02, 1.26]$, and end points at $[0.45, 0.61, 0.93, 1.08]$.

Several factors justify the choice of five discrete tempo clusters rather than a smaller number. Merging tempo clusters 4 and 5 would result in a bi-modal distribution of durations *within* the final tempo cluster. Merging clusters 1 and

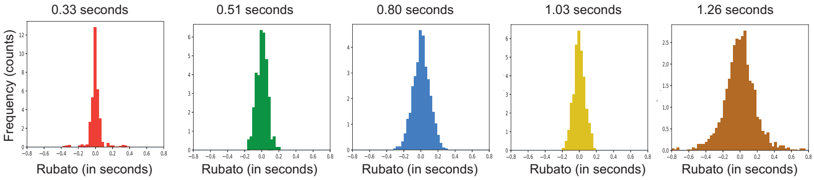

Supplementary Fig. 8 Distribution of rubato between adjacent codas for each of the 5 tempo types. The rubato for each of the tempo types is unimodally distributed around a mean value of zero.

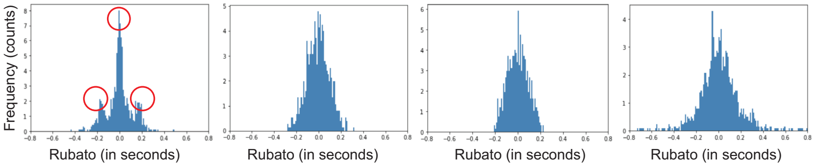

Supplementary Fig. 9 A reduction in the number of tempo clusters to four results in a multi-modal distribution of rubato.

2 causes the distribution of rubato to be bimodal only for the (new) tempo cluster 1 as shown in Fig. 9. Thus, any system with a smaller number of tempo clusters either introduces (1) a bimodal tempo distribution within the cluster or (2) a bimodal rubato distribution only within that cluster. Each of these alternatives produces a system with a single idiosyncratic cluster, in contrast to the five-cluster system.

4 Rubato

The rubato at any given point in the exchange is characterized by the difference in the durations of adjacent codas of the same rhythm and tempo type made by the same whale. The experiments below provide evidence that (1) whales gradually vary the total duration of their codas in consecutive calls, (2) gradual drifts over a period cause long-term changes in overall duration, and (3) this drift in duration is perceived and imitated by whales in the exchange.

4.1 Adjacent coda pairs have small duration differences

The durations of adjacent codas (of the same rhythm type made in consecution by a given whale) are similar, differing by an average of 0.05s. Under a null hypothesis that duration depends only on discrete coda type, and not adjacent codas, we would expect a significantly larger value of 0.08s (test: permutation test (one-sided), $p = 0.0001$, $n = 2953$, performed by randomly resampling durations within each discrete coda type without replacement).

Results are shown in the main paper Fig. 2(C).

4.2 Rubato accumulates over time, causing larger changes in duration

Changes in coda duration are positively correlated across adjacent coda triples. We compare H_0: consecutive rubato changes are uncorrelated, vs. H_1: consecutive changes in rubato are correlated and reflect longer-term trends (test: Spearman's rank-order correlation (two-sided), $r(2586) = 0.57$, $p = 2e^{-220}$, 95% CI= $[0.54, 0.60]$, $n = 2588$). Results are discussed in Section 2.1 of the main paper.

4.3 Durations of overlapping codas from different whales are imitated

For overlapping codas, the overall duration of the coda of the initiating whale is similar to the duration of the coda produced by the interlocutor: see Fig. 10. The mean observed difference in durations is 0.99s. Under a null hypothesis that chorusing whales only match discrete coda type, we would expect a significantly larger difference of 0.129 (test: permutation test (one-sided), $p = 0.0001$, $n = 908$, performed by randomly resampling durations within each discrete coda type without replacement).

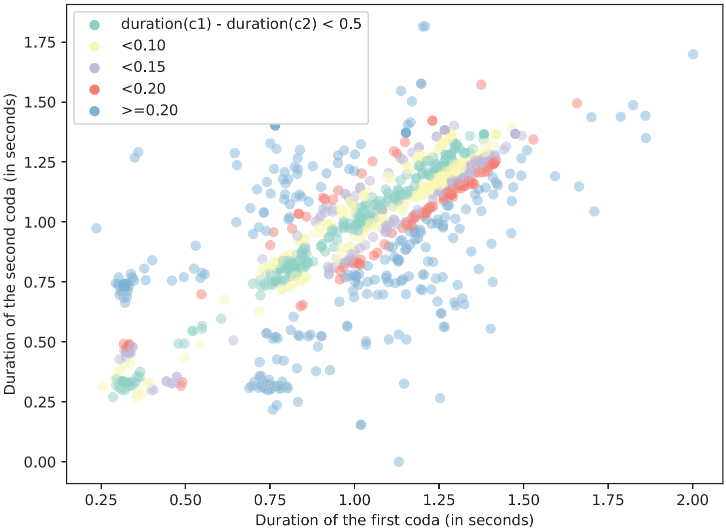

Supplementary Fig. 10 Overall durations of overlapping codas in the dataset. Overlapping codas are imitated with varying degrees of precision. The breakdown of the percentage of overlapping codas in each category and its corresponding precision range are: green: (25%) within 0.05 seconds; yellow: (45%) within 0.1 seconds; purple: (61%) within 0.15 seconds; red: (71%) within 0.2 seconds; blue: remaining set of overlapping codas (100%).

4.4 Precision of imitation

Fig. 11 (*Left*) shows the distribution of the difference in duration of overlapping codas and Fig. 11 (*Right*) shows the cumulative distribution function of the absolute difference in the duration of overlapping codas. It can be seen that (45%) of the codas' duration is imitated with a precision of less than 0.1 seconds.

When overlapping codas are made, the coda duration is determined by the leading whale, and its interlocutor has to determine the timing of its clicks before the complete coda of the leading whale can be observed. Therefore, the interlocutor has to decide when to produce the nth click (where $(n > 1)$) of its coda after the leading whale has produced only the first m clicks of its coda (where $(m > 1)$). To do this, the interlocutor is required to estimate the duration of the leading whale's coda on the basis of just the first m prefix clicks of the coda.

In (92%) of overlapped codas, the first click of the following whale is made between the first and second click of the leading whale. In (97%) of overlapped codas, the second click of the following whale is made between the second and third click of the leading whale. Estimating the duration of the leading whale's coda thus requires the second whale to both precisely measure its first ICI, and additionally infer the rhythm type of the leading whale's incompletely produced coda. Since the consecutive codas of a given whale are likely to be of the same rhythm type (see Section 2), information on the rhythm type of the leading whale is present in previously produced adjacent calls. A close matching of the overall duration by the interlocutor despite an incomplete observation of the leading whale's coda demonstrates the interlocutor's awareness of the leading whale's rhythm type.

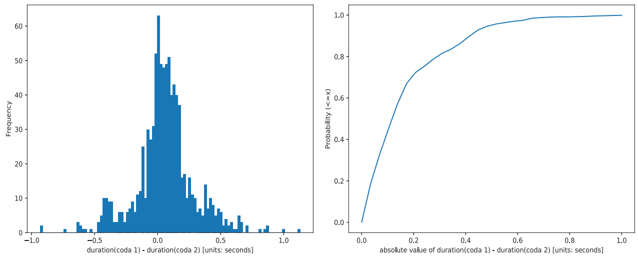

Supplementary Fig. 11 (*Left*) The difference in durations of overlapping codas is distributed tightly around zero. This indicates that the durations of codas are predominantly closely matched. (*Right*) The cumulative distribution function of the difference in durations of overlapping codas. The CDF indicates that about (70%) of the overlapping codas are matched with a precision of about $(1/5)^{th}$ of sec.

4.5 Rubato is not correlated with body motion

The same tags used to record vocalization are also instrumented with accelerometers and gyroscopes, making it possible to test for correlation

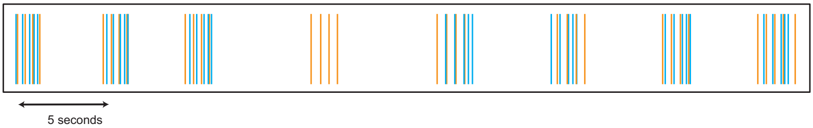

Supplementary Fig. 12 Overlapping codas made by a pair of whales in a chorus. In this exchange, the first click of the second whale in each pair of overlapping coda is made between the first two clicks of the coda of the leading whale.

between vocalization and movement. Figure 13 depicts the correlation between rubato and the orientation and acceleration of the whale recorded by sensors. We find no significant correlation. The correlation between the quantities was checked from orientation and acceleration measurements made zero seconds apart to motion readings up to 10 seconds preceding the rubato. The correlation was computed for measurements in intervals of 0.1 seconds between 0 and 10. The lack of significant correlation rules out the effect of synchronized motion being the cause of coordinated rubato change.

5 Ornamentation

Ornamented codas are anomalous in terms of the number of clicks, duration, as well as in the aspect of their rhythm from the surrounding set of codas. In this section, we provide extra information about experiments that highlight additional differences between ornamented codas and non-ornamented codas: (1) Discarding the ornament of a coda causes the coda constituted by the remaining clicks to match the rhythm type of its neighbouring codas. (2) The rubato of ornamented codas has a distribution different from non-ornamented codas. (3) ICIs of ornamented codas have anomalous statistics. Finally, (4) ornaments are not imitated by whales in a chorus.

5.1 Finding ornaments

We define an ornament as the last click of the coda that contains one more click than the immediate neighbouring codas made by the same whale. In the dataset, we find that (4%) of the codas contain an ornament.

Since ornament labels are assigned purely based on the number of clicks, the effects on rhythm, and duration described below are not necessary consequences of this procedure but constitute distinct sources of evidence for the independence of ornaments.

5.2 The first $(n-1)$ clicks of ornamented codas are more similar to neighbouring codas' rhythm type than any n-click rhythm type

Codas are grouped into rhythm types based on the number of clicks in the coda and the relative spacing of the clicks in their standardised form. However, in the case of ornamented codas, the ornament appears to be independent of the

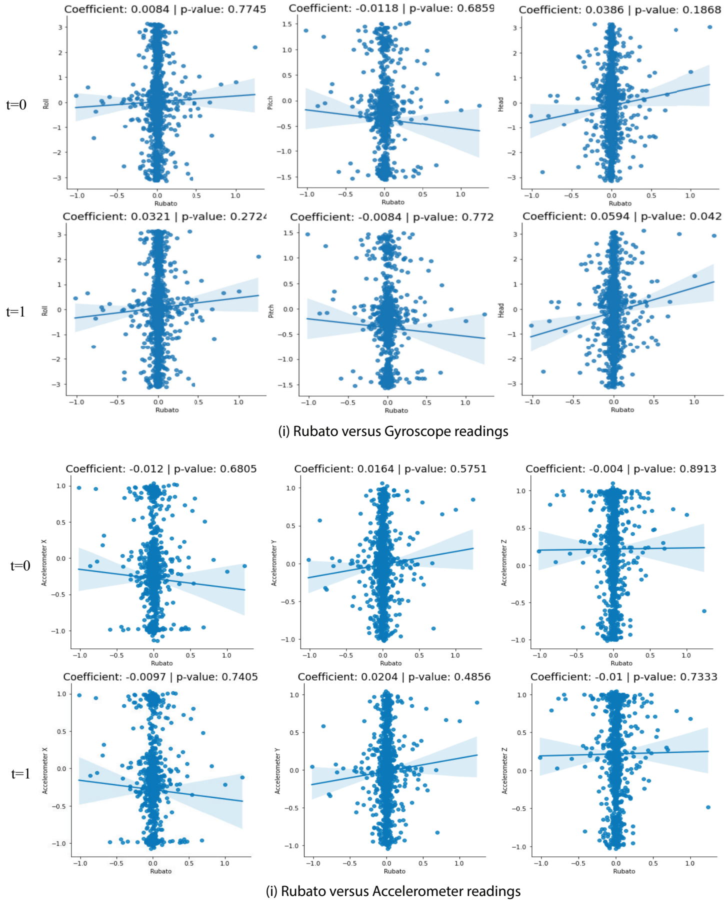

Supplementary Fig. 13 No significant correlation was found between gyroscope readings and rubato or between the accelerometer readings and rubato. The correlation between the quantities was checked from orientation and acceleration measurements made zero seconds apart to motion readings up to 10 seconds before (test: Spearman's rank order correlation test (two-sided), $n = 1172$).

rest of the clicks in the coda, and the remaining clicks together form a coda with a rhythm type matching to the neighbouring codas by the same whale. Consider a standardised ornamented coda:

$$\mathbf{c}_o = [t_2 - t_1, t_3 - t_1, t_4 - t_1, t_5 - t_1, t_6 - t_1]/(t_6 - t_1) \ . \tag{4}$$

The coda's standardised representation with the ornament-removed is:

$$\mathbf{c}_{-o} = [t_2 - t_1, t_3 - t_1, t_4 - t_1, t_5 - t_1]/(t_5 - t_1) \ . \tag{5}$$

Let `clusterCentre` denote the function that returns the cluster centroid of a standardised coda type, and let `neighbour` denote the function that returns

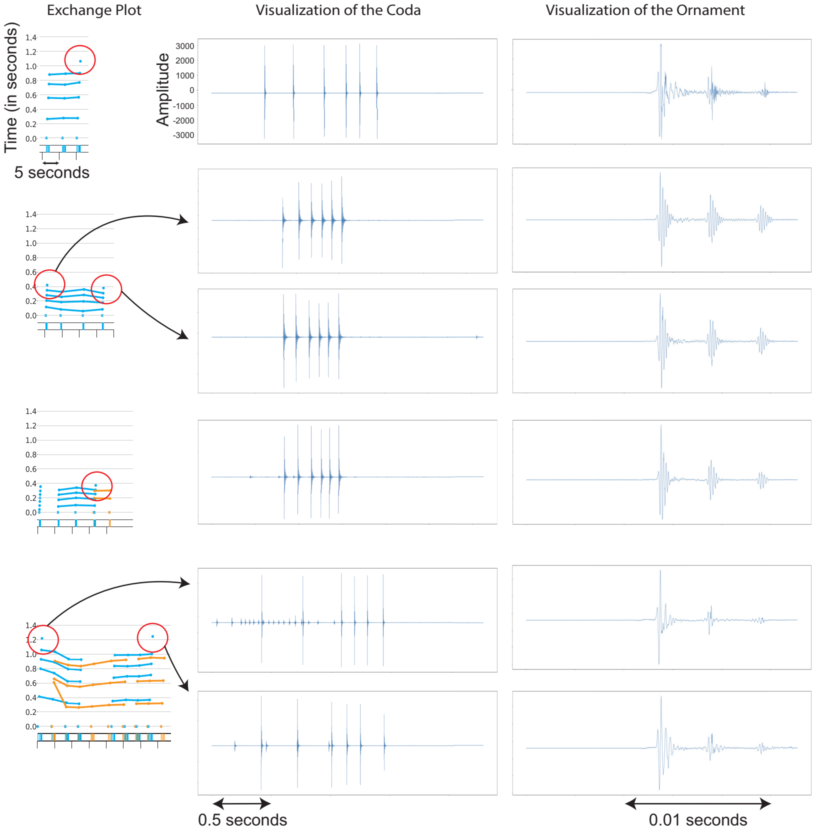

Supplementary Fig. 14 *(Left)* A snippet of an exchange plot where one of the whales produces an ornament. *(Center)* The waveform of the ornamented coda. *(Right)* The waveform of the ornament. The ornament is structurally similar to the remainder of the clicks in the coda.

a standardised neighbouring coda made by the same whale. We compare the distributions (as seen in Fig. 15):

1. Quantity 1 (in orange): $\|\texttt{clusterCentre}(\mathbf{c}_o) - \mathbf{c}_o\|_2^2$
2. Quantity 2 (in blue): $\|\texttt{clusterCentre}(\texttt{neighbour}(\mathbf{c}_o)) - \mathbf{c}_{-o}\|_2^2$

The average value of quantity 2 (0.0034s^2) is significantly less than the average value of quantity 1 ($0.0053s^2$) (test: permutation test (one-sided), $p = 0.0022$, $n = 178$, performed by randomly resampling labels [distance to cluster vs distance to neighbor's cluster]; Figure 15). Therefore, the internal distribution of clicks in an ornamented coda is more like its neighbouring codas than the coda cluster it is originally assigned to.

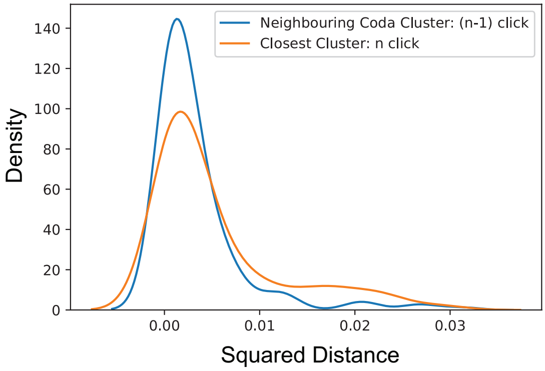

Supplementary Fig. 15 Distribution of distances of (orange) standardised ornamented codas with the standardised cluster centers of their rhythm types, and (blue) standardised ornamented codas with the ornament removed with the cluster centers of the neighbouring codas. Rhythm types of the first (n-1) clicks of ornamented codas are closer to those of the neighbouring codas than the ornamented coda's rhythm type is to its assigned cluster.

5.3 Ornamented codas have a higher variance in the difference of durations between adjacent codas by a given whale

Generally, in the course of an exchange, a whale fixes the rhythm that it makes, picks a tempo type to start with, and only gradually varies the duration of the coda. However, ornamented and non-ornamented codas exhibit significant differences in the distribution of their duration differences with neighboring codas (test: Kolmogorov-Smirnov test (two-sample), $D(206, 206) = 0.25$, $p = 5e^{-11}$, 95% CI= $[0.15, 0.35]$, $n = 206$).

5.4 ICI values of ornamented codas are atypical

As described in Section 2.1 of the main paper, the difference in the two final ICIs, normalized by the duration of the penultimate ICI, exhibits a significantly different distribution in ornamented and non-ornamented codas (test: Kolmogorov-Smirnov test (two-sample), $D(150, 3688) = 0.51, p = 1.7e^{-15}$,95% CI = $[0.42, 0.62]$, $n = (150, 3688)$; main paper Fig. 2(D)).

5.5 Ornaments are not imitated

In overlapping codas where at least one of the codas is ornamented, the number of clicks the ornamented coda is only matched (5%) of the time, compared to (71%) of the time for non-ornamented overlapping codas. The average difference in duration for overlapping codas of which at least one is ornamented (0.210s) is also significantly larger than the difference in duration of overlapping non-ornamented codas (0.099s) (test: permutation test (one-sided), p=0.0001, n=(62, 848), performed by randomly resampling labels [ornamented vs. unornamented] without replacement).

	Start of sequence	Elsewhere
Ornamented	41 (29%)	100 (61%)
Non-ornamented	594 (17%)	2893 (83%)

	End of sequence	Elsewhere
Ornamented	37 (26%)	104 (74%)
Non-ornamented	598 (17%)	2889 (83%)

	Before chorusing change	Elsewhere
Ornamented	93 (62%)	57 (38%)
Non-ornamented	1524 (51%)	1461 (49%)

Supplementary Table 1 Frequency of ornamented and non-ornamented codas at the start and end of coda sequences (top and middle respectively), and frequency before changes in vocalization style (bottom)

5.6 Ornaments are non-uniformly distributed across call sequences

If the ornamentation were an uncontrolled feature in sperm whale calls, it would be equally likely to occur anywhere in the sequence and therefore, uniformly distributed as a function of time in the call sequence. However, ornaments are more likely to be present at the beginnings and ends of call sequences. For this analysis, we consider call sequences with greater than two codas in the sequence. Fully more than half of ornaments occur at the beginning or end of a call sequence: (29.08%) in the first coda and (26.24%) in the last coda. Coda type counts by location are given in Table 1.

From this table, it can be seen that ornamented codas are more likely to occur at the start of a whale's calls compared to non-ornamented codas (test: Fisher's exact test (two-sided), odds ratio: 2.00, $p =0.0006$). Ornamented codas are also more likely to occur at the end of a whale's calls as opposed to non-ornamented codas (test: Fisher's exact test (two-sided), odds ratio: 1.71, $p =0.008$).

5.7 Ornamented codas are followed by a change in responding whale vocalizations

We define a 'change in chorusing behavior' as one of three events: a following whale begins chorusing with a leading whale, pauses chorusing, or ceases vocalizing for the remainder of the exchange. Counts by location relative to chorusing change events are also shown in Table 1. Compared to unornamented codas, ornamented codas from the *leading* whale are disproportionately succeeded by a change in chorusing behavior from the *following* whale (test: Fisher's exact test (two-sided), odds ratio: 1.56, $p = 0.009$).

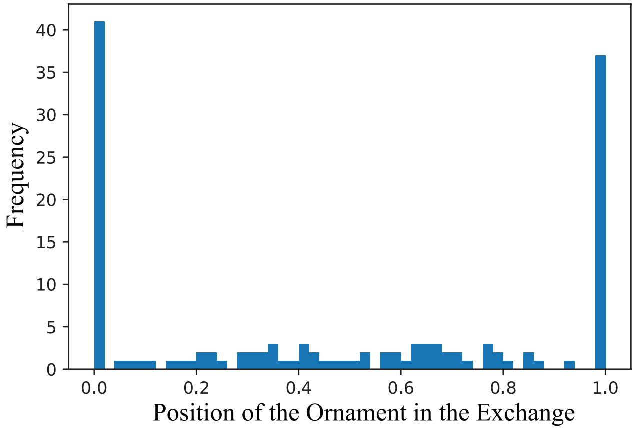

Supplementary Fig. 16 Frequency distribution of ornamented codas in the span of consecutive call sequences made by an individual whale. Calls were defined to be consecutive if they occurred no later than 8 seconds apart (which is twice the average inter-coda time as described in Figure 2 of the Supplementary Material).

**Traits in Human Language**	**Sperm Whales**
Vocal auditory channel	Yes. [2]
Broadcast transmission, and directional reception	Yes. [2, 3]
Rapid fading (Transitoriness)	Yes.
Interchangeability	Yes.
Total Feedback	Yes.
Specialization	Yes. [3]
Semanticity	Maybe. [3, 4]
Arbitrariness	Yes. [2, 3, 5–7]
Discreteness	Yes. [1, 2]
Displacement	?
Productivity	?
Traditional Transmission	Yes. [5–7]
Duality of Patterning	Maybe. [This paper]

Supplementary Table 2 Characteristics of Language: Hockett's Design Features of Natural Language. A detailed description of each of the listed features can be found in [8].

6 Information Capacity

6.1 Fixed coda inventory

Previously, the communication system of the Eastern Caribbean sperm whales was hypothesized to consist of 21 unique coda types. If at a given time a single coda is produced, such a communication system could communicate at most $\lceil \log_2 21 \rceil = \lceil 4.39 \rceil = 5$ bits per coda.

Feature	Human Language
Phonetic features	Tonality, pitch, rate, nasality, etc
Phonemes	/b/, /d/, /f/, /g/, etc
Morphemes	*eat, date, weak, -ness, -ly, -ism, etc*
Words	*eats, dated, weakness, happily, etc*
Sentences	*Lily ate some fruit.*

Supplementary Table 3 The linguistic hierarchy in humans and instances of the features in English.

6.2 Combinatorial coda inventory

The proposed system hypothesizes that codas are constructed by sampling from 18 rhythm types, the presence or absence of an ornament (2 possible values), an increasing, decreasing, or unchanging rubato (3 possible values), and one of 5 tempo types. The resulting set of codas that can be generated from such a system can contain a total of $18 \times 5 \times 2 \times 3 = 540$ symbols, capable of communicating $\lceil \log_2 576 \rceil = \lceil 9.08 \rceil = 10$ bits per coda. Therefore, the set of new features and a combinatorial coding system allows encoding a significantly greater number of messages.

However, not all possible codas are frequently realized in practice. 20 of the rhythm and tempo type combinations occur with both rubato and ornamentation, 6 with only rubato, 1 with only ornamentation, and 16 without ornamentation or rubato. Here we ignore infrequent occurrences (≤ 1 time in the DSWP dataset) of features across the different types. The resulting set of frequently generated feature combinations comprises 156 different codas, capable of communicating $\lceil \log_2 156 \rceil = \lceil 7.28 \rceil = 8$ bits per coda.

A computation based on the size of the message space alone is an upper bound on the true information rate. This is because, in practice, calls made at consecutive time steps are not independent. Consecutive calls made by a given whale are more likely to be of the same rhythm type (Section 2) and overlapping calls are likely to have the same overall duration (Section 4). Therefore, computing the information transmission capacity of the communication system requires accounting for dependencies between calls over longer time horizons, an important avenue for future work.

7 Parallels to Human Language

While an animal's communication system does not have to be human-like to exhibit non-trivial structure, human language is considered the epitome of structured communication systems due to a combination of regularity and expressivity. A comparison between the human language and sperm whale

**Linguistic Features**	**Analogues**	**Examples**
Phonetic features<br>Phonemes	Sub-coda features	Rhythm, Tempo, Rubato, Ornamentation
Morphemes / Words	Codas	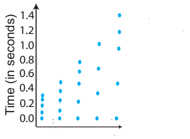
Sentences	Coda Sequences	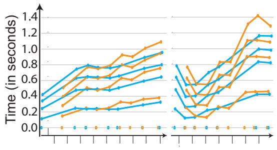

Supplementary Table 4 Analogues of the features of the linguistic hierarchy in sperm whale communication.

communication allows for benchmarking aspects of the (incompletely characterized) whale communication system against well-studied aspects of human communication. Inspired by the study by [8], Table 2 highlights the design features of natural language and what has been understood of them in the sperm whale communication system. Table 3 and Table 4 highlight features of the linguistic hierarchy in human language and their possible corresponding analogs in sperm whale communication thus far. Aspects of this characterization are provisional, and further study and analysis of the sperm whales' communication system will allow for continued in-depth characterization.

References

[1] Gero, S., Whitehead, H., Rendell, L.: Individual, unit and vocal clan level identity cues in sperm whale codas. R Soc Open Sci **3**(1), 150372 (2016)

[2] Watkins, W.A.: Sperm whale codas. J. Acoust. Soc. Am. **62**(6), 1485 (1977)

[3] Whitehead, H.: Sperm whales: social evolution in the ocean. Choice **41**(06), 41–3452413452 (2004)

[4] Whitehead, H.: Consensus movements by groups of sperm whales. Mar. Mamm. Sci. **32**(4), 1402–1415 (2016)

[5] Rendell, L.E., Whitehead, H.: Vocal clans in sperm whales (Physeter macrocephalus). Proc. Biol. Sci. **270**(1512), 225–231 (2003)

[6] Cantor, M., Whitehead, H.: How does social behavior differ among sperm whale clans? Mar. Mamm. Sci. **31**(4), 1275–1290 (2015)

[7] Gero, S., Bøttcher, A., Whitehead, H., Madsen, P.T.: Socially segregated, sympatric sperm whale clans in the Atlantic Ocean. Royal Society Open Science **3**(6), 160061 (2016)

[8] Hockett, C.D.: The origin of speech. Sci. Am. **203**(3), 88–96 (1960)
