# Supplementary material for: Contextual and combinatorial structure in sperm whale vocalisations
Source: Nat Commun. 2024 May 7;15:3617. doi: 10.1038/s41467-024-47221-8 (PMC11076547; doi:10.1038/s41467-024-47221-8)
Supplement: Supplementary file 3 — Reporting Summary [file 41467_2024_47221_MOESM3_ESM.pdf]

nature portfolio

Corresponding author(s): Daniela Rus

Last updated by author(s): Mar 5, 2024



# Reporting Summary

Nature Portfolio wishes to improve the reproducibility of the work that we publish. This form provides structure for consistency and transparency in reporting. For further information on Nature Portfolio policies, see our Editorial Policies and the Editorial Policy Checklist.

## Statistics

For all statistical analyses, confirm that the following items are present in the figure legend, table legend, main text, or Methods section.

| n/a | Confirmed | |
|---|---|---|
| ☐ | ☒ | The exact sample size ($n$) for each experimental group/condition, given as a discrete number and unit of measurement |
| ☒ | ☐ | A statement on whether measurements were taken from distinct samples or whether the same sample was measured repeatedly |
| ☐ | ☒ | The statistical test(s) used AND whether they are one- or two-sided<br>*Only common tests should be described solely by name; describe more complex techniques in the Methods section.* |
| ☐ | ☒ | A description of all covariates tested |
| ☐ | ☒ | A description of any assumptions or corrections, such as tests of normality and adjustment for multiple comparisons |
| ☐ | ☒ | A full description of the statistical parameters including central tendency (e.g. means) or other basic estimates (e.g. regression coefficient) AND variation (e.g. standard deviation) or associated estimates of uncertainty (e.g. confidence intervals) |
| ☐ | ☒ | For null hypothesis testing, the test statistic (e.g. $F$, $t$, $r$) with confidence intervals, effect sizes, degrees of freedom and $P$ value noted<br>*Give P values as exact values whenever suitable.* |
| ☒ | ☐ | For Bayesian analysis, information on the choice of priors and Markov chain Monte Carlo settings |
| ☒ | ☐ | For hierarchical and complex designs, identification of the appropriate level for tests and full reporting of outcomes |
| ☐ | ☒ | Estimates of effect sizes (e.g. Cohen's $d$, Pearson's $r$), indicating how they were calculated |

*Our web collection on statistics for biologists contains articles on many of the points above.*

## Software and code

Policy information about availability of computer code

| | |
|---|---|
| Data collection | Dataset used is available at: https://github.com/pratyushasharma/sw-combinatoriality/data |
| Data analysis | The data was analysed using custom scripts in python. The code is available at: https://github.com/pratyushasharma/sw-combinatoriality |

For manuscripts utilizing custom algorithms or software that are central to the research but not yet described in published literature, software must be made available to editors and reviewers. We strongly encourage code deposition in a community repository (e.g. GitHub). See the Nature Portfolio guidelines for submitting code & software for further information.

## Data

Policy information about availability of data

All manuscripts must include a data availability statement. This statement should provide the following information, where applicable:

- Accession codes, unique identifiers, or web links for publicly available datasets
- A description of any restrictions on data availability
- For clinical datasets or third party data, please ensure that the statement adheres to our policy

The data and code is made available under the MIT license at: https://github.com/pratyushasharma/sw-combinatoriality.

## Research involving human participants, their data, or biological material

Policy information about studies with human participants or human data. See also policy information about sex, gender (identity/presentation), and sexual orientation and race, ethnicity and racism.

| | |
|---|---|
| Reporting on sex and gender | N/A |
| Reporting on race, ethnicity, or other socially relevant groupings | N/A |
| Population characteristics | N/A |
| Recruitment | N/A |
| Ethics oversight | N/A |

Note that full information on the approval of the study protocol must also be provided in the manuscript.

# Field-specific reporting

Please select the one below that is the best fit for your research. If you are not sure, read the appropriate sections before making your selection.

☐ Life sciences ☐ Behavioural & social sciences ☒ Ecological, evolutionary & environmental sciences

For a reference copy of the document with all sections, see nature.com/documents/nr-reporting-summary-flat.pdf

# Ecological, evolutionary & environmental sciences study design

All studies must disclose on these points even when the disclosure is negative.

| | |
|---|---|
| Study description | The acoustic data used in this study was collected as a part of a longitudinal behavioural research program called The Dominica Sperm Whale Project. |
| Research sample | Our dataset includes both near-field data from 42 tags deployed on 25 different individuals in 11 different social units, with photo-identification to match the tagged whale. In addition, a large number of far-field recordings of sperm whales were also used. These made up an existing dataset of annotated codas from 2005-2018. |
| Sampling strategy | This represents one of the largest acoustic animal borne tag datasets for this species. Individuals were selected for tagging based on identity to collect samples from multiple individuals, social units, and across years. |
| Data collection | Dtag generation 3 were deployed using a hand pole by one of the authors (Shane Gero) between 2014-2018. Far field recordings were collected by the field team from The Dominica Sperm Whale Project between 2005-2018. |
| Timing and spatial scale | Recordings were collected between 2005-2018 along the western, leeward coast of Dominica in an area roughly 2000km2 |
| Data exclusions | For some of our analyses, we required the sequential timing of the codas, in this case we restricted our analyses to only the tag based recordings for which this timing was available. For all other analyses, the full dataset was used. |
| Reproducibility | *Describe the measures taken to verify the reproducibility of experimental findings. For each experiment, note whether any attempts to repeat the experiment failed OR state that all attempts to repeat the experiment were successful.* |
| Randomization | Randomization for statistical analysis was not relevant to this study. |
| Blinding | Blinding was not relevant to this study |

Did the study involve field work? ☒ Yes ☐ No

## Field work, collection and transport

| | |
|---|---|
| Field conditions | Fieldwork was conducted from small vessels at sea within several miles of the coast of Dominica. Recordings were made in both rainfall and clear days, both during daytime and night. |
| Location | Data was collected off the coast of Dominica in the eastern caribbean in an area roughly 2000km2 |
| Access & import/export | We did not collect biological samples for this study. |
| Disturbance | The acoustic tags are attached with suction cups and are considered non-invasive method of recording this species. |

# Reporting for specific materials, systems and methods

We require information from authors about some types of materials, experimental systems and methods used in many studies. Here, indicate whether each material, system or method listed is relevant to your study. If you are not sure if a list item applies to your research, read the appropriate section before selecting a response.

## Materials & experimental systems

| n/a | Involved in the study | |
|---|---|---|
| x | | Antibodies |
| x | | Eukaryotic cell lines |
| x | | Palaeontology and archaeology |
| | x | Animals and other organisms |
| x | | Clinical data |
| x | | Dual use research of concern |
| x | | Plants |

## Methods

| n/a | Involved in the study | |
|---|---|---|
| x | | ChIP-seq |
| x | | Flow cytometry |
| x | | MRI-based neuroimaging |

## Animals and other research organisms

Policy information about studies involving animals; ARRIVE guidelines recommended for reporting animal research, and Sex and Gender in Research

| | |
|---|---|
| Laboratory animals | We did not use laboratory animals. |
| Wild animals | We worked with and recorded wild sperm whales (Physeter macrocephalus). This was a non-invasive study and animals were not taken, captured, or killed. |
| Reporting on sex | We recorded predominantly adult female sperm whales and their social associates, including calves of both sexes. |
| Field-collected samples | We did not collect biological samples in the field. |
| Ethics oversight | The field protocols for approaching, photographing, tagging, and recording sperm whales were approved by either the University Committee on Laboratory Animals of Dalhousie University, Canada; the Animal Welfare and Ethics Committee of the University of St Andrews, Scotland; or Aarhus University, Denmark; and sometimes several or all of these across years in which the recordings which were used in this study were made. |

Note that full information on the approval of the study protocol must also be provided in the manuscript.

## Plants

| | |
|---|---|
| Seed stocks | N/A |
| Novel plant genotypes | N/A |
| Authentication | N/A |
